# Supplementary material for: The prevalence of pain catastrophising in nulliparous women in Nepal; the importance for childbirth
Source: PLoS One. 2024 Aug 6;19(8):e0308129. doi: 10.1371/journal.pone.0308129 (PMC11302853; doi:10.1371/journal.pone.0308129)
Supplement: S1 Appendix — (PDF) [file pone.0308129.s001.pdf]

Supplementary Information S1 Appendix

Questionnaire: Pain characteristics in Nulliparous women in Nepal

Q1. Have you previously had a pregnancy or given birth?

- Yes
- No

(If yes, thank you but this research is specifically for those women who have not experienced a pregnancy or birth.)

Q2. Have you ever had previous pain of any kind (e.g. back pain) that has lasted more than 3 months?

- Yes
- No

Q3. if the answer to the above is yes where was the pain?

Q4. Are you currently experiencing pain?

- Yes
- No

Q5. If yes what kind of pain are you currently experiencing?

Q6. If yes, How severe is your pain today, with 0 being no pain and 10 being the worst pain ever?

0 1 2 3 4 5 6 7 8 9 10

Your thoughts about pain (Pain Catastrophsing Scale PCS [16] – inserted here
Q 7. Everyone experiences painful situations…..

Q8 Do you currently have periods?

- Yes
- No

Q9. Would you consider your periods to be regular?

- Yes
- No
- Unsure

Q10 How many days in your cycle?

Q 11 Do you consider your periods to be painful?

- Yes
- No

Q 12 If yes when would you say the pain starts in relation to your period?

- Mid cycle
- 2 days before
- 1 day before
- With the menstrual flow
- After beginning the menstrual flow

Q 13 How long would you say your pain lasts during your period?

- 1 days
- 2 days
- 3 days
- The whole time the flow of you period lasts

Q 14 How intense would you say your period pain is with 0 being no pain and 10 being the worst pain ever?

0 1 2 3 4 5 6 7 8 9 10

Q 15 Do you take pain relief during your period

- Yes
- No

Q 16 Do you think your period pain impacts on your daily life? If the answer was yes, please give examples

- Yes
- No

Q17 What is your age?

- 18-22yrs
- 23-27yrs
- 28-32yrs
- 33-37yrs
- 38-42yrs
- 43-47yrs

Q18. What is your Race/ethnicity?

- Chettri
- Brahmin
- Newar
- Other

Q 19. What is your Religion?

- Hindu
- Buddhist
- Other

Q20. What is your current level of study?

- Undergraduate
- Postgraduate
- Mphil
- MRes
- Doctorate

Is there anything else about pain (of any kind) that you would like to share with us?

Thank you for taking the time to complete this survey.
