# Supplementary material for: The prevalence of pain catastrophising in nulliparous women in Nepal; the importance for childbirth
Source: PLoS One. 2024 Aug 6;19(8):e0308129. doi: 10.1371/journal.pone.0308129 (PMC11302853; doi:10.1371/journal.pone.0308129)
Supplement: Attachment — Submitted filename: PONE-D-23-39925_R.pdf [file pone.0308129.s002.pdf]

PLOS ONE

The prevalence of pain catastrophising in nulliparous women in Nepal; the importance for childbirth

--Manuscript Draft--

Manuscript Number:	PONE-D-23-39925
Article Type:	Research Article
Full Title:	The prevalence of pain catastrophising in nulliparous women in Nepal; the importance for childbirth
Short Title:	Pain catastrophising predictors in Nepalese nulliparous women
Corresponding Author:	Carol J Clark, PhD MSc PGCHE<br>Bournemouth University<br>Bournemouth, UNITED KINGDOM
Keywords:	Pain catastrophising; women's health; nulliparous; Maternal care; Nepal
Abstract:	In Lower-Middle-Income-Countries women are encouraged to present at a birthing facility for skilled care, but attending early can be associated with additional harm. Women admitted in latent labour are more likely to receive a cascade of unnecessary interventions compared with those attending a birthing facility during active labour. One reason that women present early is pain, with higher rates of admission among those who pain catastrophise. The aim of this study was to explore the prevalence of pain catastrophising in nulliparous women in Nepal and to identify predictors for pain catastrophising.<br>A prospective observational study was conducted using a semi-structured survey. The survey was completed by 170 women (18-32 years) in a higher education institution in Kathmandu. The survey included the pain catastrophising scale (PCS), current and previous pain and information about period pain, sociodemographic variables of age, ethnicity, and religion. The prevalence of pain catastrophising reported at a cut off score of PCS≥20 was 55.9% and at a cut off score of PCS≥30 was 17.1%. All women with a PCS ≥30 reported having painful periods. Those with a PCS≥20 were four times [95%CI 1.93-8.42] more likely to report painful periods affecting their daily activities ($p<0.001$) and those with PCS≥30 three times [95%CI1.10-10.53] more likely ($p<0.05$). In both cases ethnicity and age were not associated. Women with higher PCS were less likely to take pain medication.<br>A high prevalence of pain catastrophising was reported. It is important to understand how women's previous negative experiences of pain and pain catastrophising are perceived and if they are contributing to the rise in obstetric intervention, particularly caesarean births, in Nepal.
Order of Authors:	Carol J Clark, PhD MSc PGCHE
	Sujan Babu Marahatta, MSc PhD
	Vanora A Hundley, BN MSc PhD FHEA
Additional Information:	
Question	Response
**Financial Disclosure**<br><br>Enter a financial disclosure statement that describes the sources of funding for the work included in this submission. Review the submission guidelines for detailed requirements. View published research articles from *PLOS ONE* for specific examples.	Yes

Powered by Editorial Manager® and ProduXion Manager® from Aries Systems Corporation

This statement is required for submission and **will appear in the published article** if the submission is accepted. Please make sure it is accurate.<br><br>**Funded studies**<br>Enter a statement with the following details:<br>• Initials of the authors who received each award<br>• Grant numbers awarded to each author<br>• The full name of each funder<br>• URL of each funder website<br>• Did the sponsors or funders play any role in the study design, data collection and analysis, decision to publish, or preparation of the manuscript?<br><br>Did you receive funding for this work?	
Please add funding details.<br>as follow-up to "**Financial Disclosure**<br><br>Enter a financial disclosure statement that describes the sources of funding for the work included in this submission. Review the submission guidelines for detailed requirements. View published research articles from *PLOS ONE* for specific examples.<br><br>This statement is required for submission and **will appear in the published article** if the submission is accepted. Please make sure it is accurate.<br><br>**Funded studies**<br>Enter a statement with the following details:<br>• Initials of the authors who received each award<br>• Grant numbers awarded to each author<br>• The full name of each funder<br>• URL of each funder website<br>• Did the sponsors or funders play any role in the study design, data collection and analysis, decision to publish, or preparation of the manuscript?<br><br>Did you receive funding for this work?"	The funders had no role in the study design, data collection and analysis, decision to publish, preparation of manuscript<br><br>The study had no grant number as it was supported through internal quality related funding.<br>Initials of author CJC<br>Bournemouth University.<br>https://www.bournemouth.ac.uk/

Please select the country of your main research funder (please select carefully as in some cases this is used in fee calculation).<br>as follow-up to "**Financial Disclosure**<br><br>Enter a financial disclosure statement that describes the sources of funding for the work included in this submission. Review the submission guidelines for detailed requirements. View published research articles from *PLOS ONE* for specific examples.<br><br>This statement is required for submission and **will appear in the published article** if the submission is accepted. Please make sure it is accurate.<br><br>**Funded studies**<br>Enter a statement with the following details:<br>• Initials of the authors who received each award<br>• Grant numbers awarded to each author<br>• The full name of each funder<br>• URL of each funder website<br>• Did the sponsors or funders play any role in the study design, data collection and analysis, decision to publish, or preparation of the manuscript?<br><br>Did you receive funding for this work?"	UNITED KINGDOM - GB
**Competing Interests**<br><br>Use the instructions below to enter a competing interest statement for this submission. On behalf of all authors, disclose any competing interests that could be perceived to bias this work—acknowledging all financial support and any other relevant financial or non-financial competing interests.<br><br>This statement is required for submission and **will appear in the published article** if the submission is accepted. Please make sure it is accurate and that any funding	The authors have declared that no competing interests exist.

Powered by Editorial Manager® and ProduXion Manager® from Aries Systems Corporation

sources listed in your Funding Information later in the submission form are also declared in your Financial Disclosure statement.<br><br>View published research articles from *PLOS ONE* for specific examples.<br><br>**NO authors have competing interests**<br><br>Enter: *The authors have declared that no competing interests exist.*<br><br>**Authors with competing interests**<br><br>Enter competing interest details beginning with this statement:<br><br>*I have read the journal's policy and the authors of this manuscript have the following competing interests: [insert competing interests here]*<br><br>* typeset	
**Ethics Statement**<br><br>Enter an ethics statement for this submission. This statement is required if the study involved:<br><br>• Human participants<br>• Human specimens or tissue<br>• Vertebrate animals or cephalopods<br>• Vertebrate embryos or tissues<br>• Field research<br><br>Write "N/A" if the submission does not require an ethics statement.<br><br>General guidance is provided below. Consult the submission guidelines for detailed instructions. **Make sure that all information entered here is included in the Methods section of the manuscript.**	The study received ethical approval at the Institutional Review Committee of Nepal Health Co-operative Ltd. (NEHCO-IRC) at Manmohan Memorial Institute of Health Sciences (MMIHS) in Kathmandu, Nepal. NEHCO-IRC/078/589<br>A survey was administered online and paper based which could be accessed in English or Nepali. Participants who volunteered to complete the survey provided consent by completing the survey. All data collected and analysed was anonymised. Recruitment was from 15/08/2022 to 15/11/2022.

**Format for specific study types**<br><br>**Human Subject Research (involving human participants and/or tissue)**<br>• Give the name of the institutional review board or ethics committee that approved the study<br>• Include the approval number and/or a statement indicating approval of this research<br>• Indicate the form of consent obtained (written/oral) or the reason that consent was not obtained (e.g. the data were analyzed anonymously)<br><br>**Animal Research (involving vertebrate animals, embryos or tissues)**<br>• Provide the name of the Institutional Animal Care and Use Committee (IACUC) or other relevant ethics board that reviewed the study protocol, and indicate whether they approved this research or granted a formal waiver of ethical approval<br>• Include an approval number if one was obtained<br>• If the study involved *non-human primates*, add *additional details* about animal welfare and steps taken to ameliorate suffering<br>• If anesthesia, euthanasia, or any kind of animal sacrifice is part of the study, include briefly which substances and/or methods were applied<br><br>**Field Research**<br><br>Include the following details if this study involves the collection of plant, animal, or other materials from a natural setting:<br>• Field permit number<br>• Name of the institution or relevant body that granted permission	
**Data Availability**<br><br>Authors are required to make all data underlying the findings described fully available, without restriction, and from the time of publication. PLOS allows rare exceptions to address legal and ethical concerns. See the PLOS Data Policy and FAQ for detailed information.	Yes - all data are fully available without restriction

A Data Availability Statement describing where the data can be found is required at submission. Your answers to this question constitute the Data Availability Statement and **will be published in the article**, if accepted.<br><br>**Important:** Stating 'data available on request from the author' is not sufficient. If your data are only available upon request, select 'No' for the first question and explain your exceptional situation in the text box.<br><br>Do the authors confirm that all data underlying the findings described in their manuscript are fully available without restriction?	
**Describe where the data may be found in full sentences. If you are copying our sample text, replace any instances of XXX with the appropriate details.**<br><br>• If the data are **held or will be held in a public repository**, include URLs, accession numbers or DOIs. If this information will only be available after acceptance, indicate this by ticking the box below. For example: *All XXX files are available from the XXX database (accession number(s) XXX, XXX.).*<br>• If the data are all contained **within the manuscript and/or Supporting Information files**, enter the following: *All relevant data are within the manuscript and its Supporting Information files.*<br>• If neither of these applies but you are able to provide **details of access elsewhere**, with or without limitations, please do so. For example:<br><br>*Data cannot be shared publicly because of [XXX]. Data are available from the XXX Institutional Data Access / Ethics Committee (contact via XXX) for researchers who meet the criteria for access to confidential data.*<br><br>*The data underlying the results presented in the study are available from (include the name of the third party*	Complete de-identified data set is available for research purposes on application to the corresponding author.

and contact information or URL).<br>• This text is appropriate if the data are owned by a third party and authors do not have permission to share the data.<br><br>* typeset	
Additional data availability information:	

Powered by Editorial Manager® and ProduXion Manager® from Aries Systems Corporation

Manuscript with tables

Click here to access/download;Manuscript;Manuscript v2 with tables Pain catastrophising women Nepal.docx

The prevalence of pain catastrophising in nulliparous women in Nepal; the importance for childbirth

Carol J Clark[1] Sujan Babu Marahatta[2,3,4,5] Vanora A Hundley[1]

[1]Centre of Midwifery and Womens Health, Faculty of Health and Social Sciences, Bournemouth University, Bournemouth United Kingdom

[2]Manmohan Memorial Institute of Health Sciences Kathmandu, Nepal

[3]Bournemouth University, Bournemouth, United Kingdom

[4] Nepal Open University, Lalitpur Nepal

[5]Department of Public Health Sciences, University of California Davis, USA

*Corresponding author Carol Clark

Email: cclark@bournemouth.ac.uk

These authors contributed equally to this work.

Abstract

In Lower-Middle-Income-Countries women are encouraged to present at a birthing facility for skilled care, but attending early can be associated with additional harm. Women admitted in latent labour are more likely to receive a cascade of unnecessary interventions compared with those attending a birthing facility during active labour. One reason that women present early is pain, with higher rates of admission among those who pain catastrophise. The aim of this study was to explore the prevalence of pain catastrophising in nulliparous women in Nepal and to identify predictors for pain catastrophising.

A prospective observational study was conducted using a semi-structured survey. The survey was completed by 170 women (18-32 years) in a higher education institution in Kathmandu. The survey included the pain catastrophising scale (PCS), current and previous pain and information about period pain, sociodemographic variables of age, ethnicity, and religion. The prevalence of pain catastrophising reported at a cut off score of PCS≥20 was 55.9% and at a cut off score of PCS≥30 was 17.1%. All women with a PCS ≥30 reported having painful periods. Those with a PCS≥20 were four times [95%CI 1.93-8.42] more likely to report painful periods affecting their daily activities ($p<0.001$) and those with PCS≥30 three times [95%CI1.10-10.53] more likely ($p<0.05$). In both cases ethnicity and age were not associated. Women with higher PCS were less likely to take pain medication.

A high prevalence of pain catastrophising was reported. It is important to understand how women's previous negative experiences of pain and pain catastrophising are perceived and if they are contributing to the rise in obstetric intervention, particularly caesarean births, in Nepal.

Introduction

Facility births in low-and-middle-income countries (LMICs) has been encouraged over the last two decades [1] with the aim of reducing maternal and new-born morbidity and mortality [2]. Nepal has made significant progress in increasing the proportion of women accessing facilities for birth. Although this trend was reversed during the lockdown period (March-May 2020) resulting in a sharp increase in maternal mortality (24 deaths in the two-month lockdown period compared with 80 for the previous year) [3].

It might be anticipated that there would be a positive correlation between facility births and maternal and neonatal outcomes demonstrating increased quality of care. However, varying quality of care within institutions has resulted in the suggestion that facility births may not always improve outcomes [4] especially if facilities are unable to deal with obstetric emergencies. This is a particular concern is relation to early labour. Women who are admitted into hospital in the latent phase of labour (the time before labour is established) are more likely to experience unnecessary interventions compared with those admitted in the active phase of labour [5-7]. Although most of this research has been conducted in high resources settings, there is evidence that this also occurs in LMICs [8]. In these settings unnecessary intervention not only depletes scarce resources but also puts the woman at risk of increased morbidity. There is evidence in LMIC settings that women who perceive themselves to be at 'high-risk' are more likely to present to a birthing facility [4]. This perception relating to 'high risk' might be because of fear of childbirth, emotional distress, and their perceived ability to manage their pain. For example, if women know they will be offered the options of pain relief they may be more likely to seek to give birth in a facility as this may reduce their fear around childbirth [9].

It is well acknowledged that women seek admission to hospital in the latent phase of labour because they are fearful of childbirth, anxious and because they have pain [10]. Perceptions of significant pain are not new to women as many women experience discomforting pain regularly as part of their

menses (period pains). This pain can be significant enough to reduce physical and social activities as well as quality of life [11]. Painful periods are recognised by women as 'normal' and over time they build strategies to manage their pain with the knowledge that the pain will eventually subside. Painful periods have been found to be associated with an amplified pain perception response [12] and may be one factor which contributes to their pain response during labour.

Women in high resource settings report negative experiences of managing their pain at home during the latent phase of labour as they feel neglected, unsupported, and anxious [13]. As their anxiety builds it is likely women feel more pain. For nulliparous women their anxiety is further contributed to as they are not confident about when 'the right time' will be, what the birthing process will involve and when the pain will subside.

What matters most to women is to be able to experience a normal birth with good outcomes for themselves and their baby [1]. In Nepal women's abilities to manage their labour and feel in control were negatively associated with their perceptions of labour pain [14]. Pain perception involves neuropsychological processes beyond the painful sensation, these include a cognitive appraisal of the meaning of the pain which is influenced by emotion and psychophysiological and behavioural reactions [15]. Interpretation of pain can be considered on a continuum where pain perception may be considered as manageable through to completely unmanageable. For those who perceive the pain to be manageable they may be able to modulate their pain response through pharmacological treatments or non-pharmacological strategies. In contrast at the other end of the spectrum where the pain intensity is perceived as unmanageable a person may experience catastrophic thoughts about their pain. These uncontrollable negative thoughts and feelings of helplessness that some individuals experience when they feel pain is referred to as pain catastrophising. Pain catastrophising can be measured using the Pain Catastrophising Scale (PCS) [16] and more recently this has been translated and validated for use in Nepal [17]. Between country differences in pain

beliefs, coping and catastrophising but it is not clear if the findings are clinically significant, and the data presented was not gender specific [18].

Women in labour report pain catastrophising to be associated with a fear of being overwhelmed by pain, resulting in women being more likely to request pain relief [19]. It is also suggested that fear in childbirth and pain catastrophising may be driving preferences for operative births [20-22]

Pain catastrophising is not just a phenomenon of childbirth but might be considered an inherent trait associated with a history of pain experiences [23]. In a UK study of healthy students nearly half the participants reported pain catastrophising at a cut-off score of PCS ≥20 and over a fifth reported pain catastrophising at a PCS ≥30 [24]. Unsurprisingly the study showed that pain catastrophising in these young women was significantly associated with fear of pain and pain-related anxiety. In Nepal pain catastrophising has been recognised as a characteristic that influences post-operative pain intensity in addition it was reported that women were found to have significantly higher PCS scores than men and a higher PCS was correlated with increases in requirements for pain relieving medication [25]. There have been no studies exploring the prevalence of pain catastrophising in healthy young nulliparous women with a view to exploring how this might affect childbirth.

The aim of this study was to explore the prevalence of pain catastrophising in nulliparous women in Nepal and to identify predictors for pain catastrophising

Methods

Study setting

The study was conducted with one higher education institution (HEI) in Kathmandu, the capital of Nepal. The institution provides education to a range of different disciplines including nursing, pharmacy, public health, and laboratory sciences.

Study design and participants

A prospective observational study was conducted using a semi-structured survey. Data were collected in October and November 2022 using one validated questionnaire, additional questions relating to pain and demographic information with a population of nulliparous women of reproductive age.

In total, 170 women aged 18-32 years completed the questionnaire. Students were chosen as a purposive sample as they were a group the researchers had access to and they were likely to fulfil the inclusion criteria: no previous pregnancy, of reproductive age, over the age of 18 years. The questionnaire was in English and Nepali.

The study received ethical approval at the Institutional Review Committee of Nepal Health Co-operative Ltd. (NEHCO-IRC) at Manmohan Memorial Institute of Health Sciences (MMIHS) in Kathmandu, Nepal. NEHCO-IRC/078/589

A survey was administered online, and paper based which could be accessed in English or Nepali. Participants who volunteered to complete the survey provided consent by completing the survey. All data collected and analysed was anonymised. Recruitment was from 15/08/2022 to 15/11/2022.

Survey items

The questionnaire comprised the pain catastrophising scale (PCS) [16] and previous pain experiences, which were validated for use in Nepal [17]. The PCS consists of 13 items, rated on a 5-point Likert scale ranging from 0-4 with a total score ranging from 0 (no catastrophising to 52 (severe pain catastrophising). The scale has high test-retest correlation (r=0.75) across six weeks and good internal consistency of the three subscales with the total PCS (Cronbach's alpha = 0.87) [16]. The three subscales are: helplessness (questions 1-5,12), magnification (questions 6,7,13) and rumination (questions 8-11). The subscale helplessness relates to an inability to cope with pain. Magnification links with an overemphasised or amplified response to pain. While rumination refers to the negative thoughts that a person focuses on when they think of their past and present pain that evoke emotional distress. The Nepali version of the PCS (N-PCS) was also provided and reports

excellent internal consistency (Cronbach's alpha range = 0.90-0.93) [17]. Permission was sort from the Mapi Research Trust which is a non-profit organisation dedicated to improving patient outcomes [26].

In addition, participants were asked to provide information around age, ethnicity, and religion. They were asked contextual questions about previous pain and current pain and pain sites. Questions about their period pains included whether they needed to take medication for their period pains and if their period pains affected their daily activities. A Visual Analogue Scale (VAS) 0-10 was provided to report period pain intensity.

Statistical analysis

Data were collated and organised in Microsoft Excel and analysed using Statistical Package for Social Sciences (SPSS v 28). Descriptive statistics were produced, and the data were checked for normality using histograms of the score ranges. All data of total scores were normally distributed and therefore means and standard deviations were reported. Age was reported in age ranges. Ethnicity and Religion were categorised. Prevalence of pain catastrophising and analysis were categorised and carried out using two cut-off scores the first at a PCS cut off ≥ 20 and above [24,27] and at the PCS cut off ≥ 30 and above [16, 24]. The Pearson's chi square (*χ2)* test was used to test the difference in distribution between the categorical variables. We conducted Binary logistic regression to examine the associations between the independent variables age and ethnicity with cut off scores of ≥ 20 and ≥ 30 and the two dependent variables (1) painful periods; (2) painful periods affecting daily activity. Overall, 98% of the records were complete, missing data ranged from 2.4%-0% across all variables.

Results

Descriptive analysis

A total number of 170 healthy nulliparous women who were completing undergraduate degree or MSc programs completed the survey (Table 1). Most women were aged 18-22 years (89.9%) and

were Hindu (95.3%). While over 80% reported their ethnicity as either Brahmin, Chhetri or Newar. Participants' PCS scores were not associated with age, ethnicity, or religion.

Table 1 Demographic and descriptive information for study participants (N=170)

	Category	N (%)	PCS ≥20 n=75<br>*P* (*χ* 2-sided)	PCS ≥30 n=29<br>*P* (*χ* 2-sided)
Age (N=169)	18-22 years	152 (89.9%)		
	23-27 years	16 (9.5%)		
	28-32 years	1 (0.6%)	0.449	0.076
Ethnicity (N=170)	Chhetri	38 (22.4%)		
	Brahmin	65 (38.2%)		
	Newar	35 (20.6%)		
	Other	32 (18.8%)	0.684	0.959
Religion(N=170)	Hindu	162 (95.3%)		
	Buddhist	7 (4.1%)		
	Other	1 (0.6%)	0.528	0.833
PCS Pain Catastrophising Scale				

Table 2 provides a summary of descriptive data relating to the prevalence of pain catastrophising at two cut off scores PCS ≥20 and PCS ≥30. The mean PCS for all participants is reported alongside the mean scores for the three subgroups and the mean period pain intensity score.

Table 2. Pain Catastrophising Scores (PCS), prevalence of pain catastrophising and mean total PCS scores, the three subscales (Rumination, Magnification, Helplessness) period pain intensity score N= 170

**PCS recorded at two cut-offs**	**Yes N (%)**	**No N (%)**
PCS ≥20	95 (55.9%)	75 (44.1%)
PCS ≥30	29 (17.1%)	141 (82.9%)
	**Mean [SD] (range)**	
**Total PCS (range 0-52)**	18.65 [10.65] (0-44)	
**PCS Subscale scores**		
Rumination (range 0-16)	7.56 [4.58] (0-16)	
Magnification (range 0-12)	4.21 [2.87] (0-11)	
Helplessness (range 0-24)	6.88 [4.48] (0-22)	
**Period Pain intensity (range 0-10)**	6.39 [SD 2.39] (0-10)	
PCS Pain Catastrophising Scale; SD Standard Deviation		

The prevalence of pain catastrophising reported at a cut off score of PCS ≥20 was 55.9% (95/170) and at a cut off score of PCS ≥30 was 17.1% (29/170). The mean [standard Deviation (SD] PCS of the participants was 18.65 [SD 10.65] while the PCS subscales of rumination 7.56 [4.58], magnification 4.21 [2.87] and helplessness 6.88 [4.48]. The mean period pain intensity scores were 6.39 [2.39]. Pain symptoms associated with a PCS ≥20 and PCS<20 are reported in Table 3.

Table 3. Comparison of two groups PCS ≥20 and PCS<20 and reporting of period pain symptoms, current and previous pain. (N=170)

N (%)	PCS ≥20 (%)	PCS <20 (%)	*P* χ 2-sided
**Painful periods**<br><br>Yes n= 152/170 (89%)	72 (96%)	80 (84%)	
No n=18/170 (11%)	3 (4%)	15 (16%)	<0.05§
**Period Pain medication taken**<br><br>Yes n= 60/170 (35%)	32 (32%)	28 (39%)	
No n= 110/170 (65%)	67 (68%)	43 (61%)	0.074
**Period Pain affecting daily life**<br><br>Yes n=110/169 (65%)	60 (81%)	50 (53%)	
No n= 59/169 (35%)	14 (19%)	45 (47%)	<0.001
**Previous pain experiences**<br><br>Yes n= 19/168 (11%)	11 (15%)	8 (9%)	
No n= 149/168 (89%)	63 (85%)	86 (91%)	0.197
**Current pain experiences**<br><br>Yes n= 40/166 (24%)	19 (26%)	21 (23%)	
No n= 126/166 (76%)	54 (74%)	72 (77%)	0.606
§ Fishers Exact 2-sided; PCS Pain Catastrophising Scale			

Most participants reported painful periods 89% (152/170), and they were significantly more likely to have a PCS ≥20 (p <0.05) (table 3). The duration of pain during a period was reported as lasting from one day to the length of the period with the majority (45%) reporting pain lasting two days. The

percentage of participants who took pain relief was 35% (60/170) and there was a non-significant trend for those with a PCS ≥20 (p=>0.07) not to take pain relief (table 3).

Almost two thirds of participants reported that period pain affected their lives (65%) (110/169). Participants with a PCS≥ 20 were significantly more likely to report that period pains affected their daily life (p<0.001) (table 3).

A total of 19/168 (11%) reported previous pain experiences and 40/166 (24%) reported current pain experiences. The most common site of both previous pain and current pain was the low back. Neither of these variables were associated with a PCS ≥20. Pain symptoms associated with a PCS ≥30 and PCS<30 are reported in Table 4.

Table 4. Comparison of two groups PCS ≥30 and PCS<30 and their reporting of period pain symptoms and current and previous pain. (N=170)

	PCS ≥30	PCS <30	*P χ* 2-sided
**Painful periods**<br><br>Yes n= 152/170 (89%)	29 (100%)	123 (87%)	
No n= 18/170 (11%)	0 (0%)	18 (13%)	<0.05 §
**Period pain medication taken**<br><br>Yes n= 60/170 (35%)	14 (48%)	46 (33%)	
No n= 110/170 (65%)	15 (52%)	95 (67%)	0.108
**Period Pain affecting daily life**<br><br>Yes n= 110/169 (65%)	23 (82%)	87 (62%)	
No n= 59/169 (35%)	5 (18%)	54 (38%)	<0.05
**Previous pain**<br><br>Yes n=19/168 (11.3%)	6 (21%)	13 (9%)	
No n=149/168 (88.7%)	22 (79%)	127 (91%)	0.064
**Current pain**<br><br>Yes n= 40/166 (24%)	9 (32%)	31 (22%)	
No n= 126/166 (76%)	19 (68%)	107 (78%)	0.275
§ Fishers Exact 2-sided; PCS Pain Catastrophising Scale			

All women with a PCS ≥30 reported having painful periods and this association was statistically significant ($p<0.05$) (Table 4). Participants who reported that period pain did not affect their daily lives were significantly more likely to report a PCS <30 ($p<0.05$). There was no association between

those who reported period pain medication taken and a PCS ≥30 (p=0.11), or current pain and PCS ≥30 (p=0.28). There was a non-significant trend (p=0.06) showing that those with a PCS ≥30 were more likely to have had previous pain.

Interpretative Analysis

Participants with a PCS ≥20 were 5.2 times [95%CI 1.40- 19.70] more likely to report painful periods than those with a PCS of <20 (p<0.01). There were no associations between age and painful periods. Participants of Brahmin origin were 4.5 times [95% CI 1.20 – 16.88] (p<0.05) and those of Newar origin were 5.7 times [1.07 – 30.53] (p<0.05) more likely to report painful periods. All women with a PCS ≥30 reported having painful periods.

Tables 5 and 6 report factors associated with painful periods affecting the daily lives of women reporting a PCS ≥20 and PCS ≥30 respectively.

Table 5: Binary Regression exploring predictors of painful periods that affect daily life with three independent variables; PCS ≥20, ethnicity, and age N= 168

	B	S.E	Wald	df	p	Exp(B)	95% Confidence interval
PCS≥20	1.395	0.375	13.804	1	<0.001*	4.034	1.933 to 8.419
Ethnicity			3.103	3	0.376		
Ethnicity 1	-0.115	0.482	0.057	1	0.812	0.892	0.347 to 2.293
Ethnicity2	-0.668	0.535	1.656	1	0.198	0.503	0.176 to 1.433
Ethnicity 3	-0.682	0.541	1.588	1	0.208	0.506	0.175 to 1.460
Age			0.802	2	0.670		
Age 1	-0.521	0.582	0.802	1	0.370	0.594	0.190 to 1.858
Age 2	-23.086	40192.970	0.000	1	1.000	0.000	0.000
Constant	0.489	0.410	1.418	1	0.234	1.630	
Variables entered on Step 1: PCS≥20 (Pain Catastrophising Score) Baseline; **Ethnicity** baseline Chhetri, (1) Brahmin; (2) Newar; (3) Other. **Age** baseline 18-22 years; (1) 23-27 years; (2) 28-32 years. R^2 = 0.13 (Cox & Snell) 0.17 (Nagelkerke), *χ2 (6) = 22.514, p< 0.001* ** <0.05 Significant*							

Participants with a PCS≥20 were 4.0 times [95%CI 1.93-8.42] more likely to report that painful periods affected their daily activities this was statistically significant p <0.001 (table 5). Ethnicity and age were not significantly associated.

Table 6: Binary Regression exploring predictors of painful periods that affect daily life with three independent variables; PCS ≥30, ethnicity, and age N= 168

	B	S.E	Wald	df	p	Exp(B)	95% Confidence interval
PCS≥30	1.224	0.577	4.501	1	0.034*	3.399	1.098 to 10.528
Ethnicity			4.074	3	0.254		
Ethnicity 1	-0.131	0.466	0.079	1	0.778	0.877	0.352 to 2.187
Ethnicity2	-0.759	0.516	2.161	1	0.142	0.468	0.170 to 1.288
Ethnicity 3	-0.768	0.526	2.132	1	0.144	0.464	0.166 to 1.301
Age			0.470	2	0.791		
Age 1	-0.386	0.563	0.470	1	0.493	0.680	0.226 to 2.049
Age 2	-23.306	40192.970	0.000	1	1.000	0.000	0.000
Constant	0.880	0.382	5.292	1	0.021	2.411	

Variables entered on Step 1: PCS≥30 (Pain Catastrophising Score) Baseline; **Ethnicity** baseline Chhetri, (1) Brahmin; (2) Newar; (3) Other. **Age** baseline 18-22 years; (1) 23-27 years; (2) 28-32 years. R^2 = 0.073 (Cox & Snell) 0.100 (Nagelkerke), *χ2 (6) = 12.645, p< 0.05; * <0.05 Significant*

A similar pattern was seen in relation to a PCS ≥30 and painful periods (table 6). In this model participants with a PCS ≥30 were 3.4 times more likely [95%CI 1.10-10.53] to report that period pains affected their daily activities (p<0.03). Ethnicity and age were not significantly associated.

Discussion

This is the first study to report the prevalence of pain catastrophising in a group of nulliparous women from Nepal. The prevalence of pain catastrophising at a cut off PCS ≥20 was higher than a methodologically similar study carried out amongst UK university students (56% compared to 47%) [24]. In contrast nulliparous women in Nepal had a slightly lower prevalence at a cut off PCS ≥30

compared to the UK study (17% compared to 21%) [24]. This current study also explored factors that were associated with pain catastrophising. For those who reported PCS ≥20 and PCS ≥30 they were significantly more likely to have reported having painful periods, and in the PCS ≥20 group this factor was much more likely in those of Brahmin and Newar origin. Women who reported PCS ≥20 and PCS ≥30 were also significantly more likely to report painful periods which affected their daily lives, but this factor was not associated with ethnicity.

There are no direct prevalence comparisons in Nepal. A recent cohort study (n=520) reported higher median PCS scores in Nepal compared with a cohort in the United States, but the population reported on included older Nepalese women (than reported in this study) and it observed that those of Brahmin origin to be at greatest risk of developing chronic pain [28]. Worry is a central component of pain catastrophising and it is suggested that the link with chronic pain stems from the continued vigilance to the threat of the pain [29]. It is well acknowledged that many women suffer with period pains through life. It is possible that in some cultures the pain experienced on a regular cycle is re-enforced and augmented by associations with cultural myths and taboos [18,30].

Women's experiences of pain in childbirth have been reported to be associated with what they understand to be the meaning of the pain. For example, if a woman interprets her pain to be related to something productive and meaningful, she may also report being better able to manage her pain [31]. While those who associate their pain with negative thoughts are more likely to seek external support to help them manage their pain. However, it was interesting in our current study that women who had higher PCS scores were less likely to report taking pain relief medication compared with those who had lower PCS scores. This maybe because pain medication is helpful for those who have mild period pain rather than those that report more severe pain. A Cochrane review found that although non-steroidal anti-inflammatory drugs (NSAIDs) are more effective than placebo for alleviating pain, nearly half of women across the studies felt they did not achieve adequate pain

relief for their dysmenorrhea [32]. The fact that women are more likely to experience delays in receiving pain medication than men when they report abdominal pain [33] and their pain is less likely to be taken seriously [34] are some of the factors that may contribute to their pain experiences and increase their fear of pain.

Women's experiences of pain may be one factor that is influencing the change in caesarean rates that have increased threefold from 2006-2016 in Nepal [35]. Among the different ethnic groups, the Brahmin/Chhetri had a higher caesarean rate increasing from 4.4% in 2006 to 11.3% in 2016. While those of Newar/Janajati origin have increased caesarean rates from 2.3% in 2006 to 10.2% in 2016 [35]. Several authors have suggested that pain catastrophising and fear of childbirth are associated with rising rates of caesarean section [21,22]. Nulliparous women that experience fear in childbirth are three times more likely to have caesarean sections and that rate increases to fourfold for multiparous women. Although it is acknowledged that fear in childbirth may manifest during pregnancy [36], there is evidence that pain catastrophising manifests much earlier in a woman's life [24].

The caesarean section rate is greater among women with higher levels of maternal education, which may in turn be influenced by race and ethnicity [37]. In addition, weight and age have also been described as precursors to increasing rates of caesarean section [38]. We believe that pain catastrophising should also be considered as a precursor. It might be suggested that women's experiences early in life of not receiving adequate pain relief [32], coupled with the fact that pain may not be taken seriously [34] may be contributing to their negative emotions and beliefs about pain. It is suggested that there should be a commitment to train health professionals in Nepal about the importance of asking women about their previous pain experiences during their antenatal appointments. This would provide time to raise women's awareness about pain and strategies they can practice enabling them to successfully self-manage their pain in latent labour.

Study strengths and limitations

The strengths of this study are that it is the first study to explore the prevalence of pain catastrophising in nulliparous women in Nepal and to explore the association of pain catastrophising with period pains. The self-reporting nature of the study means that there may be some recall bias, although period pains are a regular occurrence and therefore this recall is frequent. The participants were from a purposeful sample of university students the majority of whom were between the ages of 18-22 years studying health related programs and so this limits the generalisability of the findings to other populations. It is recognised that there are negative attitudes towards menstruation in Nepal, but this is unlikely to have affected the responses that were anonymous. It was not the aim of the paper to address attitudes to menstruation, instead it was to understand about pain.

Conclusions

The study found a high prevalence of pain catastrophising in young nulliparous women in Nepal. Women with higher pain catastrophising scores were five times more likely to report painful periods and four times more likely to report that period pains affected their daily activities. Notably, women with higher pain catastrophising scores were less likely to take pain medication, which may be because they find pain medication to be ineffective. As women's previous negative experiences of pain relief may contribute to their fears of childbirth, it could be argued that pain catastrophising is contributing to the rise in caesarean births in Nepal. We suggest that health professionals should be educated about the multiple factors that contribute to a woman's perceptions and fears about pain through life, including their experiences of pain relief.

Acknowledgements

We would like to acknowledge the contribution of Ansha KC for her support in data collection and data entry.

References

1. World Health Organisation (WHO) 2018. WHO recommendations Intrapartum care for a positive childbirth experience 2018 1-210 [cited2023 5 July]. [Internet] Available from: https://apps.who.int/iris/bitstream/handle/10665/260178/9789241550215-eng.pdf

2. Moyer CA, Adanu RM, Engmann CM. The relationship between facility-based delivery and maternal and neonatal mortality in Sub-Saharan Africa. Int J Gynaecol Obstet. 2013;122(3):263–265. doi:10.1016/j.ijgo.2013.04.010

3. Karkee R and Morgan A. Providing maternal health services during the COVID-19 pandemic in Nepal. Lancet 2020; 8(10): E1243-1244. doi: 10.1016/S2214-109X(20)30350-8

4. Fink G, Ross R, Hill K, 2015. Institutional deliveries weakly associated with improved neonatal survival in developing countries: evidence from 192 Demographic and Health Surveys, Int J Epidemiol. 2015; 44(6); 1879-1888, doi: 10.1093/ije/dyv115.

5. Klein MC, Kelly A, Kaczorowski J, Grzybowski S. 2004. The effect of family physician timing of maternal admission on procedures in labour and maternal and infant morbidity. J Obstet Gynaecol Can 2004;26(7) 641-645

6. Lundgren I, Andren K, Nissen E, Berg M. Care seeking during the latent phase of labour – Frequencies and birth outcomes in two delivery wards in Sweden. Sex Reprod Healthc. 2013; 4(4)141-146

7. Rota A, Antolini L, Colciago E, Nespoli A, Borreli SE. Timing of hospital admission in labour: latent versus active phase, mode of birth and intrapartum interventions. A correlational study. Women Birth. 2018;31(4) 313-318 doi: 10.1016/j.wombi.2017.10.001.

8. Chuma, C., Kihunrwa, A., Matovelo, D. Mahendeka M. 2014 Labour management and Obstetric outcomes among pregnant women admitted in latent phase compared to active phase of labour at Bugando Medical Centre in Tanzania. BMC Pregnancy Childbirth. 2014; 14-68. doi: 10.1186/1471-2393-14-68.

9. Khwepeya, M., Lee, G.T., Chen, SR. Kuo, SY.2018. Childbirth fear and related factors among pregnant and postpartum women in Malawi. BMC Pregnancy Childbirth. 2018;1;391 doi: 10.1186/s12884-018-2023-7.

10. Hundley V, Downe S, Buckley S (2020) The initiation of labour at term gestation: physiology and practice implications. Best Pract Res Clin Obstet Gynecol. 202;67 4-18 doi: 10.1016/j.bpobgyn.2020.02.006.

11. Payne LA, Rapkin AJ, Lung KC, Seidman LC, Zeltzer LK, Tsao JC. Pain Catastrophizing Predicts Menstrual Pain Ratings in Adolescent Girls with Chronic Pain. Pain Med. 2016; 17(1):16-24. doi: 10.1111/pme.12869.

12. Granot M, Yarnitsky D, Itskovitz-Eldor J, Granovsky Y, Peer E, Zimmer EZ. Pain perception in women with dysmenorrhea. Obstet Gynecol. 2001; 98(3):407-411 doi: 10.1016/s0029-7844(01)01465-x.

13. Alehagen S, Wijma B, Lundberg U, Wijma K. Fear, pain, and stress hormones during childbirth. J Psychosom Obstet Gynaecol. 2005; 26(3):153–65. doi: 10.1080/01443610400023072.

14. Sapkota S, Kobayashi T, Kakehashi M, Baral G, Yoshida I. In the Nepalese context, can a husband's attendance during childbirth help his wife feel more in control of labour? BMC Pregnancy Childbirth. 2012;14:12:49 doi: 10.1186/1471-2393-12-49.

15. Garland EL. Pain processing in the human nervous system: a selective review of nociceptive and biobehavioral pathways. Prim Care. 2012;39(3):561-71. doi: 10.1016/j.pop.2012.06.013.

16. Sullivan MJ, Bishop SR, Pivik J. 1995. The pain catastrophizing scale: development and validation. Psychol Assess. 1995;7(4):524-532

17. Sharma S, Thibault, P, Abbot JH, Jensen MP. Clinimetric properties of the Nepali version of the pain catastrophising scale in individuals with chronic pain. J Pain Res. 2018;31(11):265-276 doi: 10.2147/JPR.S153061.

18. Sharma S, Ferreira-Valente A, C de C. Williams A, Haxby Abbott J, Pais-Ribeiro J, Jensen MP. Group Differences Between Countries and Between Languages in Pain-Related Beliefs, Coping, and Catastrophizing in Chronic Pain: A Systematic Review, Pain Med. 2020;1;21(9):1847-1862 doi: 10.1093/pm/pnz373.

19. Van den Bussche E, Crombez G, Eccleston C. Why women prefer epidural analgesia during childbirth: The role of beliefs about epidural analgesia and pain catastrophizing. Eur J Pain 2007; 11(3):275–282 doi: 10.1016/j.ejpain.2006.03.002.

20. Laursen M, Johansen C, Hedegaard M. Fear of childbirth and risk for birth complications in nulliparous women in the Danish National Birth Cohort. BJOG. 2009;116(10):1350-1355 doi: 10.1111/j.1471-0528.2009.02250.x.

21. Veringa I, Buitendjik S, de Miranda E, de Wolf S, Spinhoven P. Pain cognitions as predictors of the request for pain relief during the first stage of labor: a prospective study. J Psychosom Obstet Gynecol. 2011;32(3):119-125 doi: 10.3109/0167482X.2011.599898.

22. Dehghani M., Sharpe L., Khatibi A., 2014. Catastrophizing mediates the relationship between fear of pain and preference for elective caesarean section. Eur J Pain. 2014;18(4):582-589 doi: 10.1002/j.1532-2149.2013.00404.x.

23. Clark, C., Bartholomew, V., Mylod, D. and Hundley, V., 2023. The importance of pain histories for latent phase labour. Midirs Midwifery Digest. 2023;33(2);160-164.

24. Clark CJ, Kalanaviciute G, Bartholomew V, Cheyne H, Hundley VA. Exploring pain characteristics in nulliparous women; A precursor to developing support for women in the latent phase of labour. Midwifery. 2022;104:103174. doi: 10.1016/j.midw.2021.103174.

25. Subedi A, Pokharel K, Birendra Prasad Sah, Chaudhary P. Association of preoperative pain catastrophizing with postoperative pain after lower limb trauma surgery, J Psychosom Res. 2021;149:110575 doi: 10.1016/j.jpsychores.2021.110575.

26. Mapi Research Trust Mapi Research Trust: Clinical Outcome Assessments strategies (mapi-trust.org) [Accessed 20th August 2022]

27. Flink IK, Mroczek MZ, Sullivan MJ, Linton SJ. Pain in childbirth and postpartum recovery – The role of catastrophizing. Eur J Pain. 2009;13(3):312-316 doi: 10.1016/j.ejpain.2008.04.010.

28. Higgins C, Sharma S, Bimali I, Hales TG, Cameron PA, Smith BH, Colvin LA. Cross-sectional study examining the epidemiology of chronic pain in Nepal. Pain Rep. 2023;8;8(2):e1067. doi: 10.1097/PR9.0000000000001067.

29. Petrini L, Arendt-Nielsen L. Understanding pain catastrophizing: putting pieces together. Front Psychol. 2020;11:603420 doi: 10.3389/fpsyg.2020.603420 [Accessed 7 February 2023].

30. Garg S, Anand T. Menstruation related myths in India: strategies for combating it. J Family Med Prim Care. 2015;4(2):184-6. doi: 10.4103/2249-4863.154627.

31. Whitburn LY, Jones LE, Davet M-A, Small R. The meaning of labour pain: how the social environment and other contextual factors shape women's experiences. BMC Pregnancy and Childbirth. 2017;30;17(1):157 doi: 10.1186/s12884-017-1343-3.

32. Marjoribanks J, Ayeleke R, Farquhar C, Proctor M. Nonsteroidal anti-inflammatory drugs for dysmenorrhoea. Review > Cochrane Database Syst Rev. 2015;7:CD001751 doi: 10.1002/14651858.CD001751.pub3.

33. Chen EH, Shofer FS, Dean AJ, Hollander JE, Baxt WG, Robey JL, et al. Gender disparity in analgesic treatment of emergency department patients with acute abdominal pain. Acad Emerg Med. 2008;15(5):414-8. doi: 10.1111/j.1553-2712.2008.00100.x.

34. Hoffmann DE, Tarzian AJ. The girl who cried pain: a bias against women in the treatment of pain. J. Law Med. Ethics 2001;28, 13–27. doi: 10.1111/j.1748-720X.2001.tb00037.x

35. Acharya K, Paudel YR. Trend and sociodemographic corrlelates of caesarean section utilization in Nepal: evidence from demographic and health surveys 2006-2016 Biomed Res Int 2021;3:2021:8888267 doi: 10.1155/2021/8888267

36. Nieminen K, Wijma K, Johansson S, Kinberger EK, Ryding EL, Andersson G, et al. Severe fear of childbirth indicates high perinatal costs for Swedish women giving birth to their first child. Acta Obstet Gynecol Scand. 2017;96(4):438-446. doi: 10.1111/aogs.13091

37. Eliner Y, Gulersen M, Chervenak FA, Lenchner E, Grunebaum A, Phillips K, et al. Maternal education and racial/ethnic disparities in nulliparous, term, singleton, vertex cesarean deliveries in the United States. AJOG Glob Rep. 2021;4;2(1):100036. doi: 10.1016/j.xagr.2021.100036

38. Raisanen S, Gissler M, Kramer MR, Heinonen S. Influence of delivery characteristics and socioeconomic status on giving birth by caesarean section—a cross sectional study during 2000–2010 in Finland. BMC Pregnancy and Childbirth. 2014;31:14:120 doi: 10.1186/1471-2393-14-120.
