# Supplementary material for: The prevalence of pain catastrophising in nulliparous women in Nepal; the importance for childbirth
Source: PLoS One. 2024 Aug 6;19(8):e0308129. doi: 10.1371/journal.pone.0308129 (PMC11302853; doi:10.1371/journal.pone.0308129)
Supplement: Attachment — Submitted filename: Review recommendations.docx [file pone.0308129.s003.docx]

Dear editor,

Thank you very much for providing an opportunity for reviewing the article entitled “The prevalence of pain catastrophising in nulliparous women in Nepal; the importance for childbirth”. The authors are grateful for providing the information about prevalence of pain catastrophizing in nulliparous women in Nepal. It is really interesting and new for Nepal. But there are some comments which are provided directly in the text as sticky note. The article has no any line numbers, hence difficult to indicate the comments in separate pages. The authors are suggested to improve their methods and be clear in the results and discussion sections. The articles may be accepted if the authors addressed all the comments provided in the MS directly. I recommend the major revision of the article.

Thank you

Best of luck

Best regards,

Reviewer
