# Supplementary material for: The prevalence of pain catastrophising in nulliparous women in Nepal; the importance for childbirth
Source: PLoS One. 2024 Aug 6;19(8):e0308129. doi: 10.1371/journal.pone.0308129 (PMC11302853; doi:10.1371/journal.pone.0308129)
Supplement: Attachment — Submitted filename: Review 1.docx [file pone.0308129.s004.docx]

1. The topic selected is good but sample is too low to generalize it in context of Nepal. Only a college is selected in capital city of Nepal which also seems to be related to health science that often possesses a good knowledge and skill. As a prevalence study, it doesn’t indicate real status of women in colleges in local and peripheral especially rural area women. Authors are suggested to review topic and mention properly about this.
2. It is better to place keywords in alphabetical order.
3. The study design selected seems to be cross sectional but the author has mentioned prospective. Please explain in detail about design adopted.
4. In a reputed journal like Plos one, use of proper statistical language for population and sample needs to be distinguished. The author has ignored such aspects.
5. Authors are advised to keep the meaning of other attributes in demographic and other information.
