# Supplementary material for: The prevalence of pain catastrophising in nulliparous women in Nepal; the importance for childbirth
Source: PLoS One. 2024 Aug 6;19(8):e0308129. doi: 10.1371/journal.pone.0308129 (PMC11302853; doi:10.1371/journal.pone.0308129)
Supplement: Attachment — Submitted filename: May 2024 Response to reviewers.docx [file pone.0308129.s006.docx]

**Response to reviewers.**

| **Reviewers comments** | **Authors Response** |
| --- | --- |
| Reviewer 3 | Thank you for the helpful and constructive comments. Please find highlighted changes in the revised manuscript. |
| Authors need to provide an operation definition of "nulliparous women"? Still an unclear reason for choosing nulliparous women in the introduction section. | Definition of nulliparous p 4  This part of the introduction has be re-written:  The pattern of labour pain differs …….P5  and their pain management requirements. |
| How did you conform to normality? Just mentioning a histogram is not enough. Either present histogram or value of skewness. | The advice of a statistician was sought and amendments to the text p7 using histograms, Q-Q plots…. and Table 2 on p9 |
| Did the Author check the reliability of all three subscales? | The reliability of the three subscales were not explored in this paper as this was not the purpose. The subscales have been reported descriptively for context and are not used in any interpretative tests. Table 2 P9 |
| In the Introduction, the objective of the study: to explore the prevalence of pain catastrophizing in nulliparous women in Nepal and to identify predictors for pain catastrophizing.  In the Method Section, the aim of the study:  to explore perceptions of pain rather than attitudes to menstruation.  These two statements look different. can you please clarify it? | This is clarified.  The objective of the study remains as stated.  This was re-written in the methods section in response to a previous reviewer below in this reviewer/author response document *p7** and has been further clarified in the text of the paper on p7  The aim of the paper was to explore the prevalence of pain catastrophising and identify predictors for pain catastrophising rather than attitudes to menstruation. |
| The Pearson’s chi-square (χ2) test was used to test the difference in distribution between the categorical variables. What is the significance of using this study? | Statistical support was sought.  Pearson’s chi square test is being used to test if there is an association between the categorical variables. In table three for example exploring differenced between those with PCS ≥20 and PCS< 20 and reporting pain perceptions/symptoms. |
| Participants’ PCS scores were not associated with age, ethnicity, or religion. Table is one way and explaining association. Please justify it. | For clarity we have included the table and the associated text to support different readers needs. |
| There is no heading for Yes/No Response in Table 2.   6.39 [SD 2.39] (0-10)- remove SD | The heading for Table 2 has been altered to simplify on P9.  SD removed. |
| Exp(B)- Different to understand to the reader who does not have a statistical background. Some values are zero. Please consult a statistician for logistic regression.   The performance for the Logistic regression is unclear and just taking age and ethnicity.  Consult a Statistician for data analysis. | A statistician was consulted for this analysis. |
| 95% Confidence interval- 1.933 to 8.419- replace to by -. | The 95% Confidence intervals have been reported with a ‘to’ instead of – to avoid confusion with any negative numbers. |
| In conclusion "The study found a high prevalence of pain catastrophizing in young nulliparous women in Nepal". How studying one college represents Nepal. Next results are repeated in the conclusion. so the conclusion needs to be revised. | For clarity this has been re-written p 17  from one higher education institution in Kathmandu, Nepal.  The next section has been re-written p17Women with higher pain catastrophising scores…… |

| Reviewer’s comments | Authors responses |
| --- | --- |
| Reviewer 1 | Thank you for the helpful and constructive comments. Please find highlighted changes in the revised manuscript. |
| 1. The topic selected is good but sample is too low to generalize it in context of Nepal. Only a college is selected in capital city of Nepal which also seems to be related to health science that often possesses a good knowledge and skill. As a prevalence study, it doesn’t indicate real status of women in colleges in local and peripheral especially rural area women. Authors are suggested to review topic and mention properly about this. | This was a convenience sample and we have discussed the limitations and the need for further research to be able to generalise (p 18) |
| 2. It is better to place keywords in alphabetical order. | Keywords:  Maternal care, Nepal; nulliparous; pain catastrophising; women’s health |
| 3. The study design selected seems to be cross sectional but the author has mentioned prospective. Please explain in detail about design adopted | This has been amended to cross sectional as suggested. P6 |
| 4. In a reputed journal like Plos one, use of proper statistical language for population and sample needs to be distinguished. The author has ignored such aspects. | This has been amended.  P6 |
| 5. Authors are advised to keep the meaning of other attributes in demographic and other information. | This has been clarified in P 6. |
| Reviewer 2 -comments are in the text as a sticky note | Thank you for the helpful and constructive comments. Please find highlighted changes in the revised manuscript. |
| 1. Abstract should be one paragraph | This has been amended in P2. |
| In the abstract – how many Institutions | This has been clarified. In P2 |
| End of abstract – any recommendations | Recommendations added. P2 |
| Introduction 2^nd^ paragraph  It might be anticipated there would be a positive correlation between facility births and maternal and neonatal outcomes demonstrating increased quality of care (Ref) | Reference added [4] P3 |
| Add reference …As their anxiety  builds it is likely women feel more pain. For nulliparous women their anxiety is further contributed  to as they are not confident about when ‘the right time’ will be, what the birthing process will  involve and when the pain will subside. | Reference [10] added p4 |
| similar type of information is already mention in the above paragraph, hence need not to repeat  2^nd^ paragraph p 5  Women in labour report……. | This has been re-written to clarify it is referring to women in the latent phase of labour. P5 |
| Last paragraph of the introduction p 5  merge this and above pargaph. why is your study important in the health and scientific comminity? I haven't seen the importance of your study in introduction section. | Prior to reporting the aim the importance has been inserted.  P5 |
| Methods p 5  better to provide the location map where you performed study | The authors have considered the reviewer’s idea but are of the opinion that on this occasion a location map is not necessary for this study. |
| P6 how do you validate your questionnaires? who validae this? not clear in MS | This was validated by other researchers and the references have been added [16 and 17] P6&7 |
| P 6 the selection of the respondents for the questionnaires is not clear, how do you select the respondents regarding thier age, ethnicity etc? | This has been clarified. |
| P 6 have you provided questionnaires in both media (English and Nepali) as supplementary file? if so please cite here, if not provided provide the questionnaire as supplementary file as the format provided by PLoS One guideline and cite here. | This has been included in P 6  The English version is provided (S1 Appendix). |
| P6 – Sample  are you select students for questionnaires or patients? not cleat. In case of students, most of them may be unmarried and hoe do they feel about labor pain? | This has been amended and clarified.  P6 |
| Ethics p 6  This study is related to human health. I think authors should take the permission from Nepal Heath Research Council (NHRC). If you had received, pls include in methods clearly. | This has been addressed and clarified:  P6 |
| "Survey was administered online, and paper based" but who were the target groups? Students or public ot others. One source can be used by any one. Mentions clearly, how many questionnaires collected form online and how many from paper based? | This has been clarified p7 |
| P 6 Missing bracket | …This has been addressed p 6. |
| P7 Nepali version of same questions or others? | This has been clarified. P7 |
| P 7 Data of total scores were normally distributed – what is the normality? value include here. | These are included in Table 2 p 9 |
| P 7 Age was reported in age ranges | Age ranges – table 1. P8 |
| P 8 not to start the sentences by this way......  cite the table eg. ...............................(Table 2) | Re-written as suggested.  p8 |
| P 9 Table 2 2^nd^ to last line remove SD | Removed p9 |
| P9 Pain symptoms associated with a PCS ≥20 | Corrected. P10 |
| P10 Remove a full stop  Report percentages only | Removed  Only percentages reported |
| P 10 – text remove 152/170  Sentence (table 3) ‘T’ | Removed numbers – kept percentages.  (Table 3) p9, 10, 11 |
| P 11 text remove (60/170)  Sentence (table 3) ‘T’  Remove 19/168 and 40/166  In Table 4 remove ‘in’add brackets | Removed numbers and percentages kept.  ‘in’ removed  (Table 4) p11 |
| P 12 Table 4  Can you merge tables 3 and 4  Remove highlighted numbers | We tried merging the tables but thought it became confusing –  We believe it is clearer presented separately.  Highlighted numbers have been removed p12 |
| P 13 Use similar dash in the reported confidence intervals  Tables 5 and 6 report factors…. Cite the table only | Changed. P12  Amended as requested p12 |
| Table 5 SE is too high may be a defect in the calculation | The SE is high as this category only had one participant. |
| PCS≥20 | Corrected |
| Table 5 *χ2 (6) = 22.514, p< 0.001 * <0.05 Significant*  *Consistency remove italics*  Text (table 5) | Italics removed  (Table 5) p13 |
| Page 15 Table 6  PCS≥30  SE  Ethnicity | PCS ≥30  The SE is high as this category only had one participant.  In the table Chhetri is baseline, (1) Brahmin (2) Newar |
| Text  (table 6) | This has been addressed.  (Table 6) p 15 |
| P 15. Nepal harbors more than 125 ethinic people, one questions here, why you selected only these three etnics | This was a convenience sample from a Higher Education Institution.  The lack of diversity has been included as a limitation.  P 18 |
| Discussion |  |
| P 15 (56% compared to 47%) discuss clarification why and how | This has been re- written and clarified.  p14, |
| P 16  (17% compared to 21%)  whats the reason behind it? necessay to discuss, discussion isnot the collection of literature. Justify your answer clearly | This has been re-written to address the comments  P15 |
| Why not to other community? Justify | See above – this has been re-written p15-16 |
| your study is only focused on three ethnicity. how can you say painful period was not associated witj ethnicity. | This has been re-written for clarification  P16 |
| Those of Brahmin origin to be at greatest risk of developing chronic pain [28]  why than other ethnic groups need to elaborate? | This has been elaborated P15,16 |
| It is possible that in some cultures the pain experienced on a regular cycle is re-enforced and augmented by associations with cultural myths and taboos [18. 30]  What type of cultures associated? Need to elaborate | This has been elaborated. P15, 16 |
| P 17 The fact that women are more likely to experience delays in receiving pain medication than men when they report abdominal pain and their pain is less likely to be taken seriously [34] are some of the factors that might contribute to their pain experience and increase their fear of pain  What type of factors | Re- written for clarification  P 16 |
| Among the different ethnic groups,  the Brahmin/Chhetri had a higher caesarean rate increasing from 4.4% in 2006 to 11.3% in 2016.  While those of Newar/Janajati origin have increased caesarean rates from 2.3% in 2006 to 10.2% in  2016 [35]. Several authors have suggested that pain catastrophising and fear of childbirth are  associated with rising rates of caesarean section [21,22].  what are the major reasons that Brahmin/Chhetri had a higher caesarean rate than other ethnic. These results may come due to sampling bias. As many questionnaires were supplied to the Brahmin and Chhetri than other ethnic groups | This has been re-written across two paragraphs, P17 |
| The caesarean section rate is greater among women with higher levels of maternal education which may in turn be influenced by race and ethnicity [37] .  *justify your results here, you said in the results " Ethnicity and*  *age were not significantly associated." this statement is just opposite than your results...* | This section has been rewritten keeping the focus on the theme relating to women’s pain experiences continues. Therefore references [37, 38] have been removed from the text p17, and the reference list p21 |
| The participants were from a purposeful sample of university students the majority between the ages of 18-22 years ….  *age of the respondents is contradictory, are you sampling the age group between 18-22 or 18-32?* | To improve clarity this section has been re-written.  P17 |
| It was not the aim of the paper to address attitudes to menstruation, instead it was to understand about pain.  *Have you clearly addressed this term in the method section* | *We have addressed this in the methods section p7,8 |
| *in some cases medical persons (especially private hospital) also forced to patients for caesarean births than normal birth. have you found any virw from your questionnaires? if so include this in resuts and discussion section.*  I haven't seen any articles from PLoS. follow the guideline of PLoS | No views were sought or captured in relation to caesarean sections.  PLoS guidelines are being followed |
| *Doi missing* | Addressed p20 |
| Sapkota S, Kobayashi T, Kakehashi M, Baral G, Yoshida I. In the Nepalese context, can a  husband’s attendance during childbirth help his wife feel more in control of labour? BMC Pregnancy  Childbirth. 2012;14:12:49 doi: 10.1186/1471-2393-12-49.  *Check one time* | Addressed p21 |
| Whitburn LY, Jones LE, Davet M-A, Small R. The meaning of labour pain: how the social  environment and other contextual factors shape women’s experiences. BMC Pregnancy and  Childbirth. 2017;30;17(1):157 doi: 10.1186/s12884-017-1343-3.  *Follow the format* | Addressed p 22 |
